# ChemSpectra: a web-based spectra editor for analytical data

**DOI:** 10.1186/s13321-020-00481-0

**Published:** 2021-02-10

**Authors:** Yu-Chieh Huang, Pierre Tremouilhac, An Nguyen, Nicole Jung, Stefan Bräse

**Affiliations:** 1 Institute of Biological and Chemical Systems-Functional Molecular Systems (IBCS-FMS), Karlsruhe Institute of Technology, Hermann-von-Helmholtz-Platz 1, 76344 Eggenstein-Leopoldshafen, Germany; 2 Institute of Organic Chemistry, Karlsruhe Institute of Technology, Fritz-Haber-Weg 6, 76131 Karlsruhe, Germany

**Keywords:** Spectroscopy, Analysis, NMR, IR, Mass spectrometry, JCAMP-DX

## Abstract

ChemSpectra, a web-based software to visualize and analyze spectroscopic data, integrating solutions for infrared spectroscopy (IR), mass spectrometry (MS), and one-dimensional ^1^H and ^13^C NMR (proton and carbon nuclear magnetic resonance) spectroscopy, is described. ChemSpectra serves as web-based tool for the analysis of the most often used types of one-dimensional spectroscopic data in synthetic (organic) chemistry research. It was developed to support in particular processes for the use of open file formats which enable the work according to the FAIR data principles. The software can deal with the open file formats JCAMP-DX (IR, MS, NMR) and mzML (MS) proposing these data file types to gain interoperable data. ChemSpectra can be extended to read also other formats as exemplified by selected proprietary mass spectrometry data files of type RAW and NMR spectra files of type FID. The JavaScript-based editor can be integrated with other software, as demonstrated by integration into the *Chemotion* electronic lab notebook (ELN) and *Chemotion* repository, demonstrating the implementation into a digital work environment that offers additional functionality and sustainable research data management options. ChemSpectra supports different functions for working with spectroscopic data such as zoom functions, peak picking and automatic peak detection according to a default or manually defined threshold. NMR specific functions include the definition of a reference signal, the integration of signals, coupling constant calculation and multiplicity assignment. Embedded into a web application such as an ELN or a repository, the editor can also be used to generate an association of spectra to a sample and a file management. The file management supports the storage of the original spectra along with the last edited version and an automatically generated image of the spectra in png format. To maximize the benefit of the spectra editor for e.g. ELN users, an automated procedure for the transfer of the detected or manually chosen signals to the ELN was implemented. ChemSpectra is released under the AGPL license to encourage its re-use and further developments by the community.

## Background

The online and interactive visualization of spectroscopic data is crucial for modern scientific work to be able to evaluate scientific data and to analyze it. Web-based solutions are beneficial because of their platform-independent use and few system requirements. As web-based software developments usually can be embedded into manifold projects, they may serve as a valuable contribution to existing databases and information systems (like electronic lab notebooks, ELNs, or repositories). In chemistry research, in particular the information from ^1^H and ^13^C NMR, IR, and mass spectroscopic experiments is of high importance as these four techniques are essential for the identification of molecules. Due to their significance, they generally form the standard set of analytical data that has to be provided along with the scientific publication of synthetic results. NMR, MS and IR data can be analyzed either manually from printed spectra or they can be analyzed in detail using commercial software or free stand-alone tools. The available commercial or non-open source software products usually include manifold functionalities for processing, visualization, analysis and documentation but they have to be installed and work in most of the cases in a non-embedded manner. Examples for such software options are MestreNova [1], ChemAxon [2], TopSpin (NMR) [3] or Spectragryph (IR) [4]. Besides professional tools as the given ones, only a few web-based developments are available as an Open Source. For the spectra types NMR, MS and IR which are considered in this work, the web-based visualization tools JSpecView [5, 6], NMRPro [7], MetaboAnalyst [8], MetaboHunter [9], COLMAR [10], jsNMR [11], and SpeckTackle [12] are known. Some of them are already integrated into web services, e.g. SpeckTackle is used in MetaboLights for NMR/MS data [13]. Other databases such as the databases Human Metabolome Database HMDB [14] or DrugBank [15] are supported by additional editors that are developed internally explicitly for those databases [16].

Due to the need for advanced spectra editors for the visualization but also for the analysis of spectroscopic data such as NMR, MS and IR data with peak-picking, NMR signal integration, coupling constant calculation and multiplicity assignment, we initiated a project that is based on currently available source code and tools from own developments. The aim of the development is the extension of the applicability of web-based editors to enable their use for enhanced data management tasks in particular for web-based data management systems such as ELNs and repositories.

## Implementation

ChemSpectra consists of three modules, the react-spectra-editor, chem-spectra-client, and chem-spectra-app. For the client-side implementations (react-spectra-editor and chem-spectra-client) the software is written in the programming language JavaScript using the framework React.js which offers a user-friendly single page application to upload, read, edit, and download spectra. JavaScript was chosen due to its benefits for interactive web tools to facilitate the embedding of the developments into other web-based software. The jcampconverter library [17] inside the react-spectra-editor extracts spectroscopic data of the composed JCAMP-DX file which is sent from the server-side.

The server-side spectra handling (chem-spectra-app) is based on python and was built using a modified version of NMRglue [18] and SciPy [19]. Python as a backend programming language on the framework Flask provides data processing and ensures the compatibility with and re-use of the previously developed systems.

**Fig. 1:**
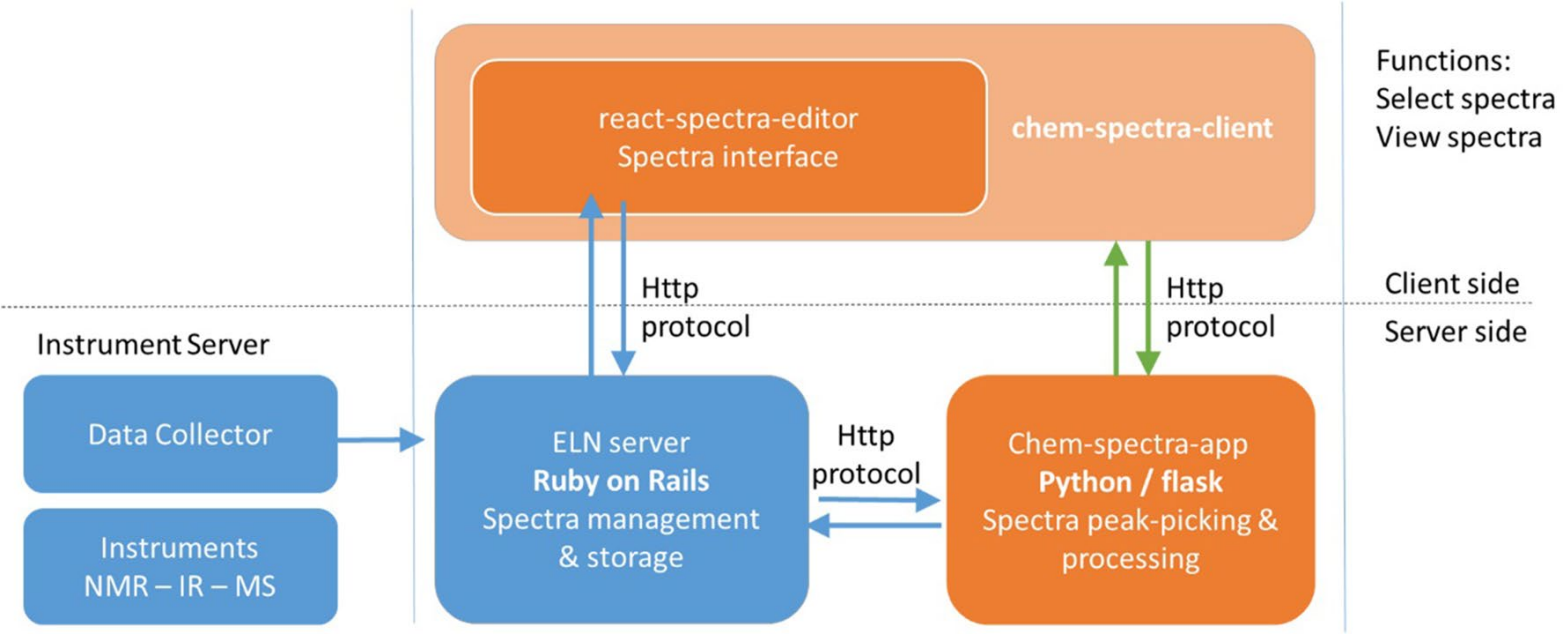
General scheme describing the overall implementation. Orange = necessary parts for a stand-alone version of ChemSpectra, blue: implementation to *Chemotion* ELN and connection of analysis results via an instrument server

ChemSpectra can be used as stand-alone software to be offered as an independent web service or can be used for other web developments. The stand-alone application supports the visualization and analysis of spectra, but its functions are limited in comparison to an embedded version, as the information stored in the browser exists temporarily and is not persisted permanently. To show the advantages of the ChemSpectra development as an embedded application offering the full available functionality, it was incorporated into the web applications *Chemotion* ELN and repository, which are developments of our research group reported earlier [20, 21]. Examples for further functions are the storage and management of the files that were edited by ChemSpectra. Figure 1 gives an overview of the main parts of the ChemSpectra software and the implemented processes for the stand-alone version (green arrows) and the exemplary implementation with the *Chemotion* applications (workflow given in blue). While, for the stand-alone implementation, the chem-spectra-app communicates directly with the chem-spectra-client, the communication for the embedded software is managed by the server of the ELN or repository. If ChemSpectra is embedded into other web applications, further systems and work processes such as a data provider can be added to the overall workflow. In the herein depicted example, an instrument server that provides analytical data that were collected by a data collector [22] of the ELN is connected.

ChemSpectra has been optimized and was tested thoroughly on the Browser Chrome. A stand-alone server version and a version of ChemSpectra embedded into the *Chemotion* ELN and repository are available for demonstration at the *Chemotion* project website, www.chemotion.net. In addition, the source code of the project for chem-spectra-app [23], chem-spectra-client [24], and react-spectra-editor [25] can be retrieved from github. The source code is released as an Open Source under the license AGPL version 3.

## Results

**Fig. 2:**
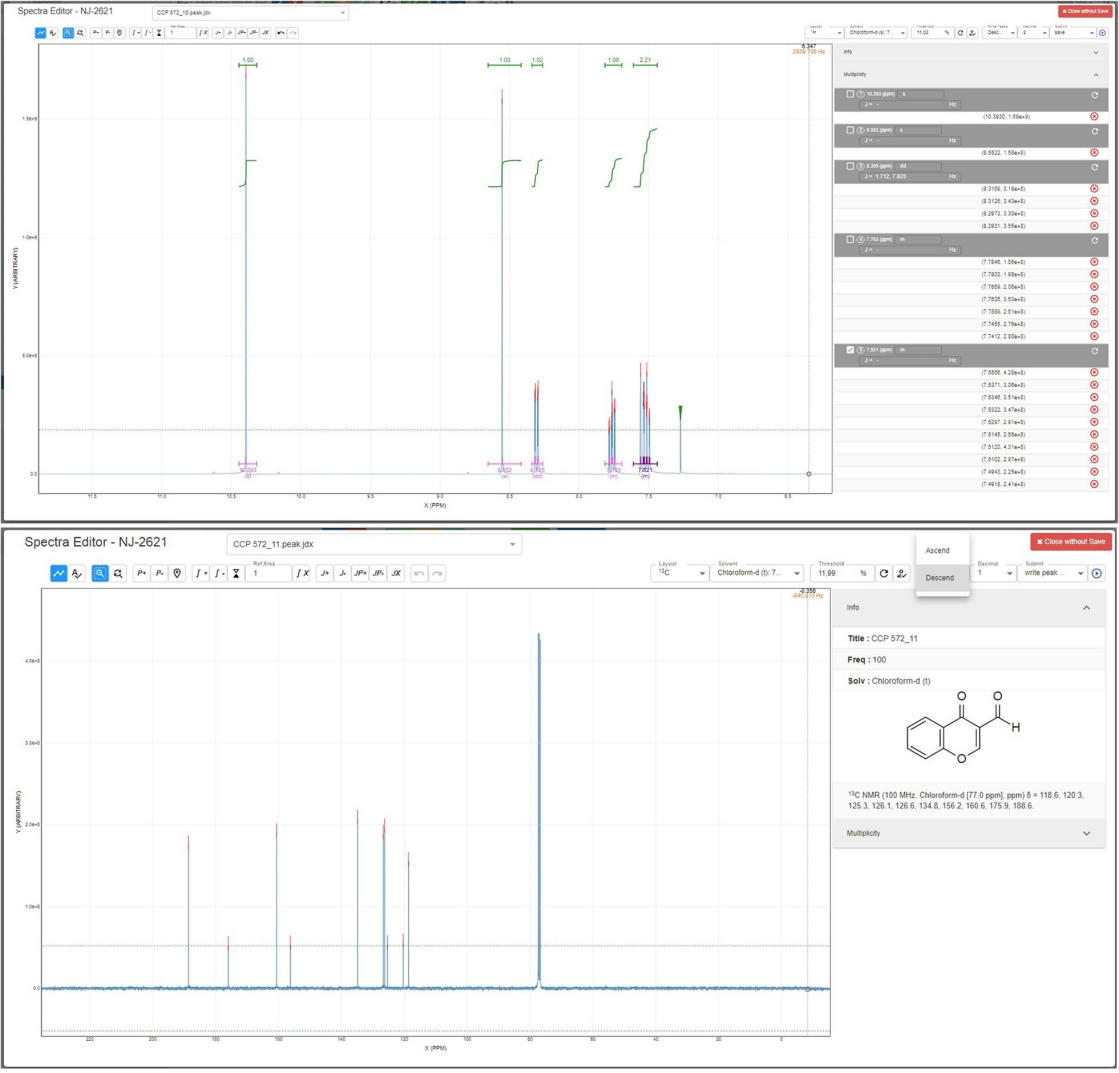
UI of the react-spectra-editor for ^1^H NMR data (upper part) and ^13^C NMR data (lower part) generated for a commercial sample of 4-oxo-4*H*-chromene-3-carbaldehyde measured in CDCl_3_. Additional information such as the generated peak lists or the information on the structure of the assigned sample is shown in the right information panel. The main toolbar for basic edit functions is given above the spectra visualization panel

**Fig. 3:**
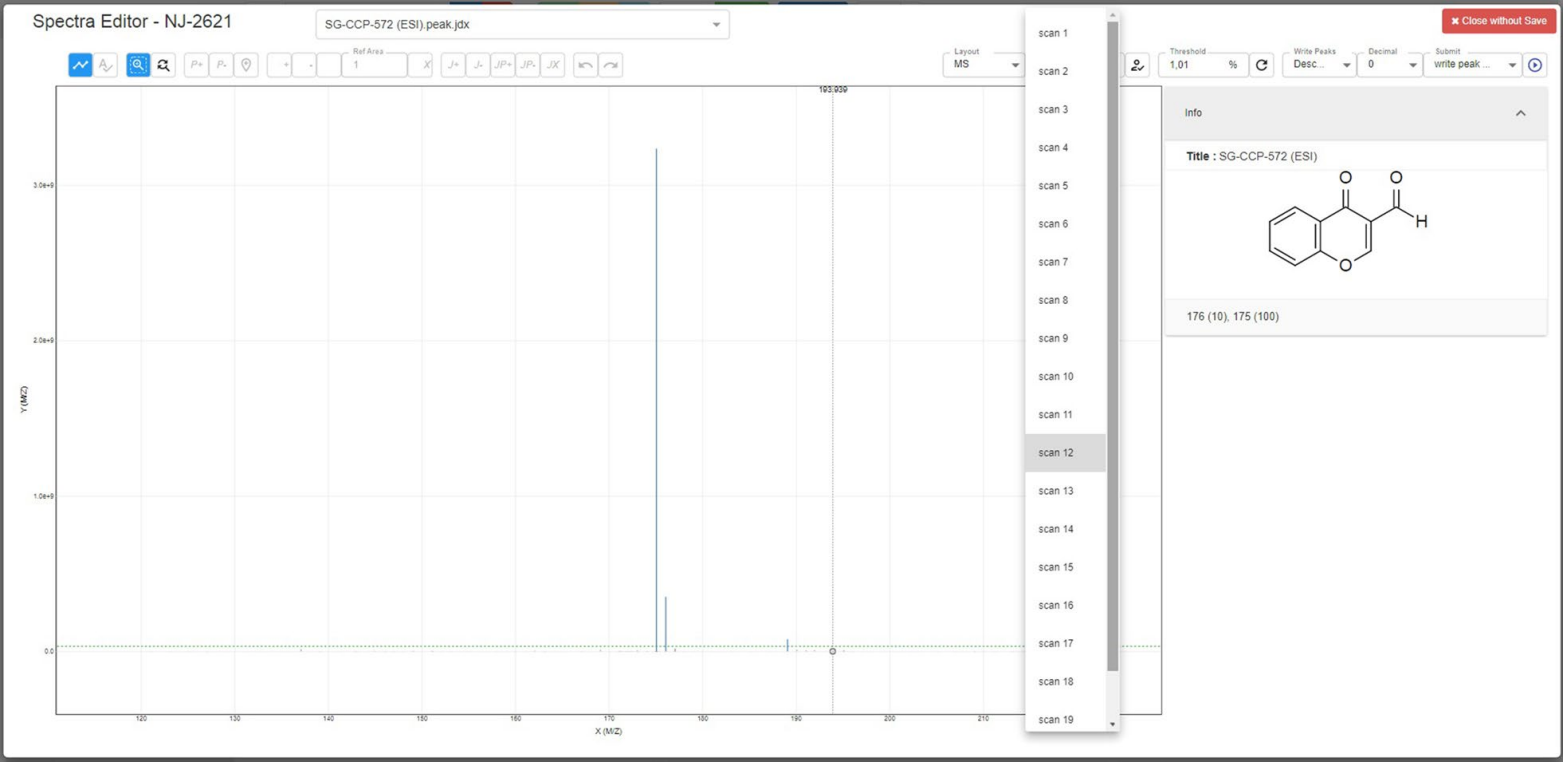
Overview of the react-spectra-editor for mass spectrometry data applied to the file format RAW that was recorded with a ThermoFisher Modell QExactive Plus mass spectrometer for commercial 4-oxo-4*H*-chromene-3-carbaldehyde. The selection options for the available scans are shown

The main part of the ChemSpectra software is the react-spectra-editor which displays the three types NMR, MS and IR data. The type of spectra is extracted automatically from the provided files. Depending on the extracted type, one of two layouts available for visualization is used: the line plot (NMR and IR data) or the bar graph (MS data). To edit the provided data with ChemSpectra, a control panel offers generic and data-type specific actions to analyze and configure the given data (Figs. 2 and 3). The generic actions are available for NMR, IR and MS data and allow to (1) zoom in and out (2) adjust the threshold that is given as default for each spectra type, and (3) extract the peaks and write them in a list form. With respect to the selected signals in the spectra, the user can select the number of displayed digits for each signal and in which order the signals should be summarized (descending or ascending).

The backend part of the ChemSpectra software, the chem-spectra-app, manages the decoding and composing of spectra files, the peak-picking, and the image generation as basic parts for the transformation of the given spectra. Currently, the chem-spectra-app accepts the file extensions jcamp, jdx, and dx for NMR, IR and MS spectra, mzML [26] and RAW files for MS spectra and FID or ZIP files for NMR spectra. If ChemSpectra is integrated into a work environment such as an ELN or repository server, the chem-spectra-app is a microservice that is in charge of all spectra-related processes, excluding storage and management (which are the main requests gated by the host web application).

### Spectra editor and control panel for one dimensional ^1^H NMR and ^13^C NMR data

As the different analysis types need different actions to edit the corresponding data, ChemSpectra enables analysis-specific actions in the react-spectra-editor UI. In the case of NMR data, these specific actions are the addition and/or removal of peaks, integration of signals, coupling constant and multiplicity calculation and assignment. Multiplicities are automatically inferred by known libraries [27, 28] and are checked by additional rules to ensure the correctness of the results. The generated information such as the identified signals, coupling constants and multiplicity can be summarized in form of a signal list. Additionally, the ChemSpectra editor offers a list of the most common reference solvent shifts for ^1^H and ^13^C NMR spectra, allowing the correction of the values given by default.

### Main and control panel for IR data

**Fig. 4:**
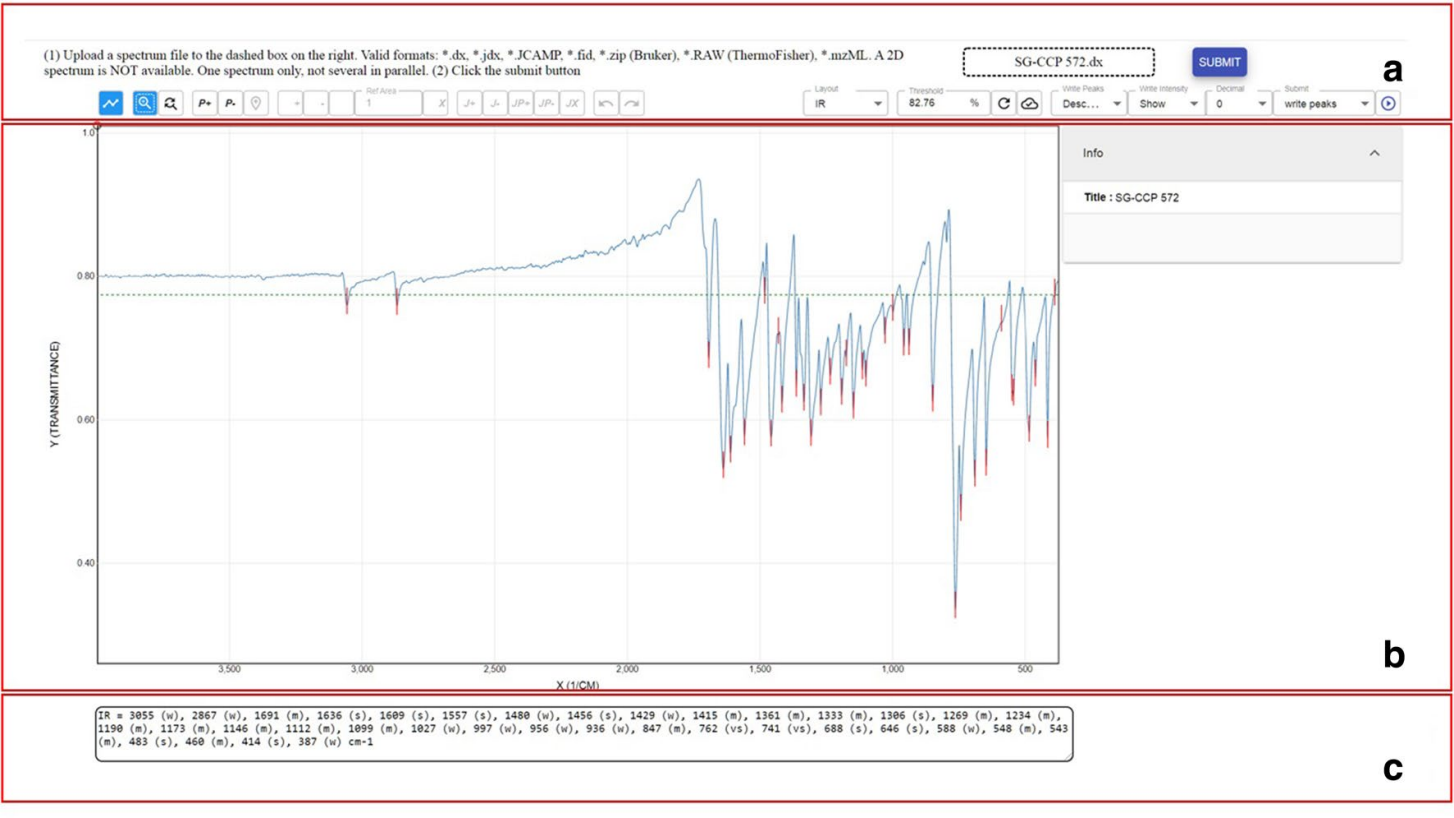
The ChemSpectra UI as the stand-alone version used for the visualization of an IR spectrum of commercial 4-oxo-4H-chromene-3-carbaldehyde, uploaded as JCAMP-DX (*.dx) file. Highlighted are the UI parts **a** file upload and user input panel for the analysis of spectra, **b** react-spectra-editor and additional information to view the results and **c** text output panel. The notifications are not shown

The IR editor and control panel offer the general three functions given for all types of spectra: adding and removing peaks, including an overview of the added and removed signals, and additionally an option to extract the given signals. Corresponding to the reporting standards for IR spectroscopy, the intensity of the identified signals (vw, w, m, s, vs) can be added to the wavenumber that is recorded. The current implementation gives the information in brackets after the corresponding wavenumber (see Fig. 4 for IR spectra in the stand-alone software).

### Main and control panel for mass data

Mass spectra differ from NMR and IR spectra in that way, that they may consist of different scans for one measurement. Depending on the internal procedure of an institution, mass spectra—if they are provided digitally—are either provided as original files including all scans that were measured, or are provided as one preselected scan. The ChemSpectra control panel for mass spectrometry offers therefore a dropdown menu including a list of the scans that are provided with each file. Per default, the first scan is visualized by the editor, but the user can change this setting to any scan that is more suitable for the analysis. Figure 3 illustrates the functions of ChemSpectra for mass spectra with the example 4-oxo-4*H*-chromene-3-carbaldehyde. The threshold line can be used to select the signals and the unselected peaks become grey to be clearly distinguishable from the selected ones. Individual signals can be selected to show the m/z value and intensity of the signal. The example shown in Fig. 3 was gained with the file format RAW (recorded with the ThermoFisher instrument QExactive Plus) demonstrating that the editor can be adapted to read and process also proprietary file formats. With respect to the FAIR data principles, data storage and further processing of proprietary file formats is not a preferred or desired procedure but in some cases, alternatives to the use of proprietary files are currently missing. Therefore, spectra editors should offer options to cover this need if possible. ChemSpectra was used to read data files of two instruments of ThermoFisher [model QExactive Plus (ESI) and Thermo Finnigan Mat 95 (EI and FAB mode)] as test-cases for data that are not given in an open file format. Since the RAW file format contains binary data, it has to be decoded before the processing with ChemSpecta is possible. For this purpose, msConvert in Proteowizard [29] is employed to convert MS files from RAW to mzML. MsConvert in a docker container is called by the chem-spectra-app to achieve this job. mzML files are converted to JCAMP-DX using pymzML, an Open Source python mass spectrometry file parser.

### Stand-alone version

In its stand-alone version, ChemSpectra runs with an additional UI—the chem-spectra-client—that allows the user to add input or retrieve output as an alternative to a connection of other resources or target systems. The chem-spectra-client UI provides three functions in addition to the react-spectra-editor: (1) a file management to upload the data to be visualized/analyzed, (2) notifications to the user and (3) a text output that is generated to be copied for further use of the generated data (Fig. 4).

### Embedded implementation

**Fig. 5:**
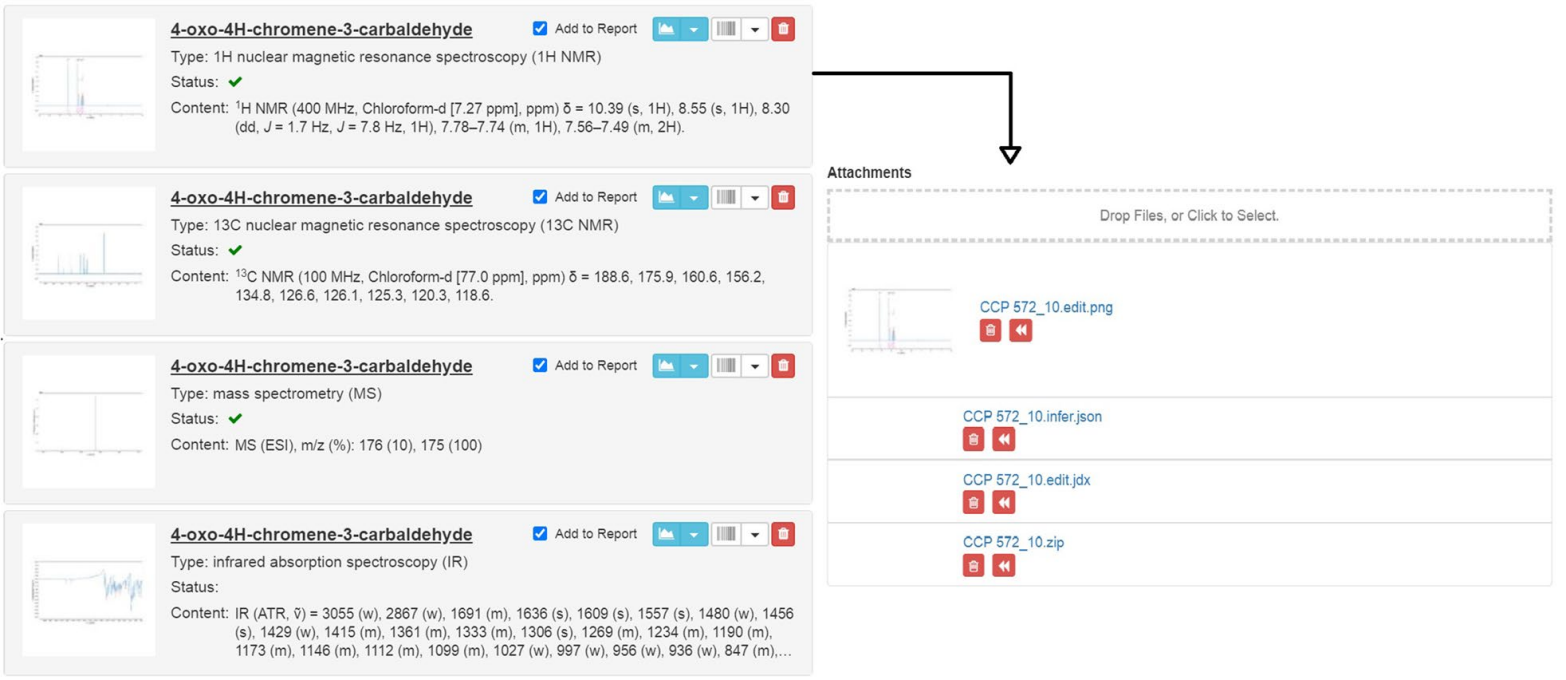
Embedded ChemSpectra in *Chemotion* ELN: visualization of data preview in the analysis section of the ELN given for ^1^H and ^13^C NMR, MS and IR data (left panel). The peak lists can be transferred directly from react-spectra-editor UI to the ELN. The right panel gives an overview of the automatically generated file types for one uploaded ^1^H NMR zip file (including a processable fid file) provided by a Bruker 400 MHz instrument

Depending on the desired interactions, the implementation with another web application such as an ELN or repository requires additional efforts for system-specific adaptations. For an implementation with *Chemotion* ELN, which is described here exemplarily, different work processes of the ELN have to be merged with ChemSpectra including the direct use of files that were generated from analytical instruments. In addition, challenges such as data persistence, supporting the storage of data for the full data life cycle, and a workflow management need to be considered. In this regard, embedding ChemSpectra into the *Chemotion* ELN was realized by keeping the original input files and adding the newly composed files as persisted data. The original files stored without any modifications are an important resource for any future referencing issues, while the composed files are kept to avoid the need for repeated analyses. Additionally, two images are generated: a low-resolution thumbnail for preview and a higher resolution for the reuse for example in publications. Both images are regenerated every time a user edits a spectrum. The embedding of the ChemSpectra editor results in the availability of a set of file formats that can be generated fully automatically without the input of the user or edited if further actions are desired. Figure 5 shows with the example of an ^1^H NMR spectroscopy file, how the implementation with the analysis section of *Chemotion* ELN is realized, giving three relevant files for the user: an original file (*.zip), a user-edited version (*.edit.jdx) and an image file (*.edit.png) [30]. A direct benefit of the implementation of ChemSpectra with an ELN is the transfer of the gained data analysis to the ELN. This allows the fast analysis of spectroscopic data and the fast and error-free documentation of the obtained results.

## Discussion of the limitations of the current developments

ChemSpectra was developed as a basis to reach independence from commercial software for standard analytical measurements. The software allows the integration with other web-developments and offers flexibility to cover further analytical techniques with forthcoming extensions. In the current version, the focus of the developments lay on the definition of basic functionality that covers the most important needs for NMR, IR and MS spectra analysis and the design of a model for smart integration to web-based information systems. The software does not offer a comprehensive solution to special types of measurement yet and lacks certain functions compared to established and specialized software. Considering for example NMR analyses in Organic Chemistry, further improvements of the editor should contain functions for the viewing and analysis of 2D data and functions for the comparison of different spectra in one application window. Additionally, the processing of FID files has to be improved by adding advanced phase correction and baseline correction methods (see Additional files 1 for an example). For mass spectrometry analysis, the chem-spectra-app should be extended to support further MS file formats. Initiatives like OpenChrom [31] show how extensive but also successful such a project is when the given challenges are to be solved by the community. The compatibility of ChemSpectra with additional file types and also types of analytical measurements will be an important extension of ChemSpectra in the future, building a framework for interoperable analytical data and its use in full compliance to FAIR principles.

## Conclusion

ChemSpectra is a software to swiftly visualize and analyze analytical data, integrating solutions for IR (infrared spectroscopy), mass spectrometry (MS), and one-dimensional ^1^H and ^13^C NMR (proton and carbon nuclear magnetic resonance) spectroscopy data. It serves as a decentralized work-instrument for the analysis of the most often used types of spectroscopic data in synthetic (organic) chemistry research, being able to deal with the open file formats JCAMP-DX (IR, NMR, MS) and mzML (MS). The software is offered as an Open Source to allow the further extension to other file formats by the community as exemplarily shown for mass spectra files of the type RAW and NMR spectroscopy files of type FID gained from common analytical instruments. All data files that are provided as non-JCAMP-DX files are processed and converted to JCAMP-DX, allowing a standardized treatment of all data files after a first processing step. ChemSpectra is provided in two versions, as a standalone version to be used as an independent service and as an integrated editor for the *Chemotion* web applications electronic lab notebook (ELN) and repository. The embedded ChemSpectra editor allows the storage of the original spectra along with edited versions, the automatic peak detection according to a default or manually defined threshold and the storage of an automatically generated image of the spectra in png format. To maximize the benefit of the embedded editor for users, a workflow to write the automatically detected or manually chosen signals was implemented. This allows the direct transfer of information to e.g. the ELN or repository. ChemSpectra consists of different modules that are used to build the core software (chem-spectra-app and react-spectra-editor) and the necessary extensions for its use as stand-alone service (chem-spectra-client). As exemplified with the *Chemotion* ELN and repository implementation, it can be adapted to other work environments. ChemSpectra should serve as a basic software to be extended in the future with respect to further data type-specific analysis functions and its usability for additional file formats. ChemSpectra is released under the AGPL license to encourage its re-use and further developments by the community.

## Supplementary Information

**Additional file 1.** The Supporting Information contains further documentation on the transformation of spectra with ChemSpectra, Flow charts for processing spectra of different file formats, decoding and parsing of mass spec, NMR and IR data files. Also, the communication overview with the ELN environment is given for the ELNembedded ChemSpectra software. Examples for processing of JCAMP-dx files and fid files in comparison are given.

## Data Availability

The Additional file contains further documentation on the transformation of spectra with ChemSpectra, Flow charts for processing spectra of different file formats, decoding and parsing of mass spec, NMR and IR data files. Also, the communication overview with the ELN environment is given for the ELN-embedded ChemSpectra software. Examples for processing of JCAMP-dx files and fid files in comparison are given. Project name: ChemSpectra. Project homepage: eln.chemotion.net. Project demo page: https://eln.chemotion.net/chemspectra-editor. Additional videos are deposited here: https://github.com/ComPlat/react-spectra-editor. Operating system(s): platform-independent access, developed/tested on Linux and Mac, deployed on Linux. Library requirements (for developer): (1) user interface: react-spectra-editor (dependencies: React.js [32]; jcampconverter [33], D3.js [34] (jcamp processing in JavaScript). (2) Peak-picking and jcamp processing: chem-spectra-app (and dependencies: flask (web server) [35], nmrglue (spectra reading) [36], pymzML [37] (mzML to JCAMP-DX conversion), pyopenms [38] (mzXML to JCAMP-DX conversion), netCDF4 [39] (cdf to JCAMP-DX conversion), matplotlib (image processing), numpy [40], and scipy (peak-picking) [41]. (3) Coupling constant and multiplicity inference: cheminfo-js/spectra [30]. (4) RAW to mzML conversion: docker and msConvert in Proteowizard [29], numpy [32], scipy [19, 41] and Matplotlib [42]. Other requirements: Modern internet browser supporting HTML5 and JavaScript. Recommended browsers: Chrome. Programming language: JavaScript, Python. Source Code on Github: Chem-spectra-app (https://github.com/ComPlat/chem-spectra-app) chem-spectra-client (https://github.com/ComPlat/chem-spectra-client), and react-spectra-including demos and videos (https://github.com/ComPlat/react-spectra-editor) can be retrieved from github. Source Code on Zenodo: chem-spectra-client: 10.5281/zenodo.4059273; react-spectra-editor 10.5281/zenodo.4059278; chem-spectra-app 10.5281/zenodo.4059263. License: AGPLv3.

## Abbreviations

ELN: Electronic Laboratory Notebook
UI: User interface
JCAMP-DX: Joint Committee on Atomic and Molecular Physical data extension
NMR: Nuclear magnetic resonance
IR: Infrared
MS: Mass spectrometry
AGPL: Affero General Public License
